# Bioinformatic Insights into the Carotenoids’ Role in Gut Microbiota Dynamics

**DOI:** 10.3390/nu18020330

**Published:** 2026-01-20

**Authors:** Helena R. Rocha, Pedro Ribeiro, Pedro Miguel Rodrigues, Ana M. Gomes, Manuela Pintado, Marta C. Coelho

**Affiliations:** Universidade Católica Portuguesa, CBQF—Centro de Biotecnologia e Química Fina—Laboratório Associado, Escola Superior de Biotecnologia, Rua Diogo Botelho 1327, 4169-005 Porto, Portugal; mhrocha@ucp.pt (H.R.R.); s-pmsbribeiro@ucp.pt (P.R.); pmrodrigues@ucp.pt (P.M.R.); amgomes@ucp.pt (A.M.G.); mpintado@ucp.pt (M.P.)

**Keywords:** carotenoids, dietary antioxidants, oxidative stress reduction, provitamin A activity, nutritional status, gut microbiota, fermentation dynamics, metagenomic analysis, hierarchical clustering

## Abstract

**Background/Objectives:** Carotenoids are bioactive pigments with well-established antioxidant and immunomodulatory properties, yet their impact on gut microbiota remains poorly understood from a chemical standpoint. This study explores how carotenoid structure and gastrointestinal stability shape microbial responses combining in vitro fermentation with bioinformatic analyses. **Methods:** Individual carotenoids (beta (β)-carotene, lutein, lycopene) and combined carotenoids, as well as algal-derived extracts were subjected to 48 h in vitro fermentation, and microbial composition and activity were assessed through sequencing and computational analysis. **Results:** β-carotene and lycopene promoted acid-tolerant taxa such as *Escherichia-Shigella*, whereas lutein, due to its higher polarity, supported more transient fluctuations. Mixtures and algal carotenoids exhibited synergistic effects, sustaining beneficial genera including *Bifidobacterium* and *Bacteroides* and promoting structured ecological trajectories. **Conclusions:** These findings provide a chemistry-driven perspective on how carotenoids act as modulators of microbial ecosystems, with direct implications for the formulation of carotenoid-enriched functional foods and dietary interventions.

## 1. Introduction

**Figure 1:**
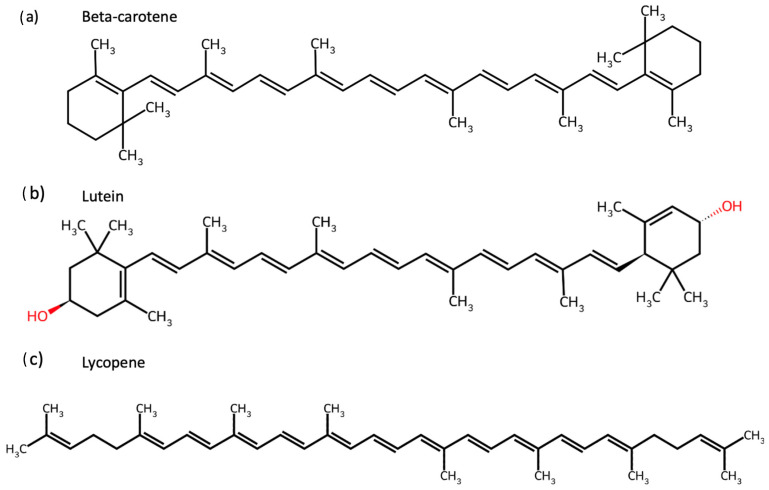
Chemical structures of carotenoids tested individually in this study. Structures of (**a**) Β-carotene, (**b**) lutein, and (**c**) lycopene were obtained from ChemSpider and evaluated for their effects on gut microbiota composition and activity.

Carotenoids are isoprenoid-based pigments widely distributed in fruits and vegetables, whose structural diversity—including cyclic end-groups, conjugated double bonds, and hydroxyl substituents—critically determines their solubility, oxidative stability, and biological activity [1,2,3,4]. Traditionally recognised for their role in human nutrition, these compounds, such as beta(β)-carotene, lutein, and lycopene, exhibit potent antioxidant, anti-inflammatory, and immunomodulatory properties, contributing to disease prevention and health promotion [5,6]. In particular, these biological activities are strongly linked to mechanisms relevant to non-communicable diseases (NCDs), including oxidative stress, chronic low-grade inflammation, and impaired metabolic regulation [7,8,9,10,11,12,13,14]. Among the many available carotenoids are beta-carotene, lutein, and lycopene, each of which was targeted individually in this study, and their chemical structures are shown in Figure 1. The alga *Osmundea pinnatifida* contains additional carotenoids, including zeaxanthin and beta-cryptoxanthin [15], which were not tested individually but may contribute to the observed effects of the algal extract.

The intestinal microbiota (IM), often referred to as the “second genome”, comprises a vast and complex community of microorganisms that perform essential physiological functions, including aiding digestion, regulating metabolism, synthesising vitamins, modulating immune responses, and maintaining the intestinal barrier. The composition and activity of the IM are highly sensitive to dietary influences and imbalances, termed dysbiosis, and have been associated with multiple health conditions such as obesity, type 2 diabetes, inflammatory bowel disease, and neurodegenerative disorders [5,6,15]. Given the central role of dysbiosis in the onset and progression of major NCDs, dietary compounds capable of restoring microbiota balance have emerged as promising modulators of disease risk [16,17,18,19,20,21]. This intricate interplay between diet, microbiota, and health has catalysed growing interest in identifying dietary components, such as carotenoids, that can beneficially modulate microbial ecosystems [4,15].

Carotenoids exhibit their effects through multiple mechanisms. Following digestion, they are metabolised into bioavailable forms that can interact with the gut microbiota [6]. Recent advancements, such as the INFOGEST standardised in vitro model, have provided insights into how carotenoids interact with gut microbial communities under physiologically relevant conditions. Notably, Rocha et al. (2024) [15] demonstrated that carotenoids can modulate microbial composition by increasing the relative abundance of beneficial taxa like *Lachnospiraceae* (~77.8%) while decreasing potentially pathogenic genera such as *Enterococcus* (−16.3%), *Streptococcus* (−8.8%). These changes align with microbial profiles associated with improved gut health [15]. Furthermore, carotenoids stimulate the production of organic acids (OAs) such as succinic, acetic, butyric, and propionic acids, which are critical for maintaining gut barrier integrity, reducing inflammation, and supporting overall intestinal homeostasis. Such metabolite shifts are particularly relevant because disturbances in gut barrier function, inflammation, and microbial metabolism are key contributors to NCD pathophysiology [22,23,24,25]. These bioactive metabolites act as signalling molecules that promote crosstalk between microbiota and host cells, modulating immune and metabolic pathways [26,27].

Within the gastrointestinal tract, carotenoids undergo isomerisation, oxidation, and enzymatic cleavage, generating bioactive metabolites whose activity depends on structural features such as polarity and conjugation length. These molecular properties influence their solubility, aggregation behaviour, and interactions with bile salts, digestive enzymes, and microbial consortia [4,6].

Despite these advances, the underlying mechanisms through which carotenoids influence microbial dynamics remain only partially understood. Comprehensive investigations using cutting-edge tools are essential to unravel these processes. Bioinformatics offers a transformative approach to exploring these complex interactions, enabling in-depth analyses of metagenomic and metabolomic datasets. By identifying specific microbial taxa and metabolic pathways influenced by carotenoids, bioinformatics provides insights into the microbial networks that contribute to gut health. Moreover, such analyses support the development of personalised nutrition strategies, as they consider individual microbiota compositions and dietary preferences [28].

Building upon the findings of Rocha et al. (2024) [15], the present study focuses specifically on the metagenomic dimension of carotenoid-microbiota interactions. Using 16S rRNA sequencing and bioinformatic analysis of relative abundance data, we investigate how individual carotenoids, a carotenoid mixture, and an algal-derived matrix modulate the taxonomic structure of the IM following simulated gastrointestinal digestion. Unlike previous work that included metabolite profiling [15], the current study was designed exclusively to characterise compositional shifts, providing a targeted assessment of microbial responses at the taxonomic level. This metagenomic approach contributes to a clearer understanding of how carotenoids influence microbial community structure and supports future developments in functional foods and dietary strategies.

## 2. Materials and Methods

### 2.1. Preparation of Carotenoids and Algal Samples

To maintain the stability of carotenoid samples during digestion, a solution with 1 mL of polysorbate 20 M (Tween 20) and 19 mL of phosphate-buffered solution (PBS) at pH 7.4 was added to all samples to prevent rapid degradation. The beta(β)-carotene solution contained 11.9 mg of β-carotene, 19 mL of PBS, and 1 mL of Tween 20 (0.57 mg/mL). Lutein and lycopene solutions included 500 μL of their respective carotenoid and 2.5 mL of Tween 20 (lutein at 1.00 mg/mL; lycopene at 2.50 mg/mL). The mixed carotenoid solution (MIX) combined 500 μL each of β-carotene, lutein, and lycopene with 1.5 mL of Tween 20 (6.00 mg/mL). For *Osmundea pinnatifida* (Praia da Agudela, Portugal), 3 g of the alga was homogenised using a kitchen robot (Bimby TM6, Vorwerk, Wuppertal, Germany) and suspended in 3 mL of Tween 20. The carotenoids’ concentrations used in the in vitro digestion and fermentation experiments were selected primarily to ensure chromatographic detectability and method reproducibility, rather than to mimic dietary, physiological intake levels. This approach was necessary due to the high susceptibility of carotenoids to degradation during digestion and to guarantee sufficient analytical sensitivity for high-performance liquid chromatography with diode-array detection (HPLC-DAD, Agilent) quantification. Although these concentrations do not correspond to recommended dietary doses, they enabled the robust assessment of carotenoid stability, release, and transformation throughout the experiments.

### 2.2. In Vitro Method for Simulating Gastrointestinal Digestion

Carotenoid solutions underwent simulated gastrointestinal digestion using the INFOGEST 2.0 protocol described by Brodkorb et al. [29]. Each sample was tested alongside a negative control (PBS). The process began with an oral phase where 3 mL of the carotenoid solution was mixed with 2.4 mL of simulated salivary fluid (SSF) containing CaCl_2_ and α-amylase (84 U/mg of powder; A1031-5KU; Sigma-Aldrich, St. Louis, MO, USA), incubated at 37 °C and 200 rpm for 2 min (Orbital Shaker MaxQ 6000). Subsequently, 4 mL of simulated gastric fluid (SGF) with CaCl_2_ and rabbit gastric extract ((resuspended rabbit gastric extract lipase and pepsin at 15 U/mg of powder; Lipolytech, Marseille, France)) was added and incubated at 37 °C and 130 rpm for 2 h to simulate gastric digestion. The intestinal phase was followed by adding 4 mL of simulated intestinal fluid (SIF) with CaCl_2_, bile salts (0.2 g/mL; bile extract porcine-B8631; Sigma-Aldrich), and pancreatin (6 U/mg of powder; P7545; Sigma-Aldrich), incubated at 37 °C and 45 rpm for 2.5 h. To terminate enzymatic activity, the digested samples were immediately cooled on ice before being dialysed against water using 3.5 kDa membranes (pre-wetted RC Tubbing, Spectra/Por^®^6 Dialysis Membrane; 734–0652; VWR Chemicals, Radnor, PA, USA) at 37 °C and 50 rpm overnight to evaluate absorption potential. Aliquots were collected after each digestion step (oral, gastric, and intestinal phases) to assess the carotenoid availability for absorption both within and outside the dialysis membrane. All digestions were performed in triplicate to ensure reproducibility.

To further evaluate digestion efficiency, the recovery indexes were calculated whenever analytically feasible. The recovery index represented the overall stability of the compounds throughout digestion and was obtained by comparing the total carotenoid content recovered at each stage of the digestion with the initial amount introduced before the oral phase. These parameters were determined only for samples in which chromatographic peaks remained well resolved, quantitatively detectable, and above the method’s limit of quantification. In some groups, carotenoid quantification was not possible throughout the entire digestion, as some compounds were rapidly degraded, isomerised, or adsorbed to digestion components, causing their concentrations to fall below the detection limit during the gastric or intestinal phases. For this reason, recovery indexes could only be calculated for the groups in which the parent carotenoids remained measurable (Table 1).

### 2.3. In Vitro Faecal Fermentations

The in vitro fermentation methodology started with collecting fresh faecal samples from five healthy donors, complying with rigorous ethical standards outlined by the Health Ethics Committee of Universidade Católica Portuguesa (project n° 167). Donors, healthy volunteers in Porto, Portugal, three men and two women aged between 23 and 39 years, were free from chronic diseases, allergies, recent probiotic or prebiotic consumption, and antibiotic usage within the preceding six months. Each participant provided informed consent according to the Declaration of Helsinki guidelines. Faecal samples were promptly transferred to sterile vials and maintained anaerobically for a maximum of 2 h before processing.

The faecal inoculum preparation involved diluting the faecal matter to a concentration of 100 g/L in reduced physiological salt (RPS) solution (0.5 g/L cysteine hydrochloride-Merck, Darmstadt, Germany) containing cysteine hydrochloride and NaCl (LabChem, Zelienople, PA, USA), achieving a pH of 6.8 within an anaerobic workstation (Don Whitley Scientific, West Yorkshire, UK). Homogenisation was facilitated using a stomacher (Serward, Worthing, UK) operating at 460 paddle beats per minute for 2 min [15,30].

Faecal samples from the five donors were pooled in equal proportions to prepare a composite inoculum for the fermentations. This pooling strategy provided a representative microbiota for assessing the effects of carotenoid treatments while minimising interindividual variability. Fermentations were performed in triplicate using this pooled inoculum. Individual donor variability was not assessed in this study.

For the fermentation medium, a blend was created comprising tryptone soya broth (TSB) without dextrose (Fluka Analytical, St. Louis, MO, USA), bactopeptone (Becton Dickinson Biosciences, Franklin Lakes, NJ, USA), yeast nitrogen base, cysteine hydrochloride (Merck, Darmstadt, Germany), and specific salt solutions (A and B) (Merck, Darmstadt, Germany) adjusted to pH 6.8. Trace mineral (ATCC, Manassas, VA, USA) and resazurin (Sigma-Aldrich Chemistry, St. Louis, MO, USA) solutions were also added to monitor the fermentative process. Fructooligosaccharides (FOS; Nutripar, Matosinhos) were included as a positive control at 2% (*m*/*v*). Carotenoid samples recovered from colon fractions after dialysis were added to respective fermentation flasks at a final concentration of 2% [15,30].

Following inoculation with faecal material at a concentration of 2% (*v*/*v*), the sterilised fermentation flasks were sealed and incubated at 37 °C for 48 h within an anaerobic atmosphere composed of 10% CO_2_, 5% H_2_, and 85% N_2_. Sampling occurred at 0, 6, 12, 24, and 48-h intervals during incubation, with pH levels measured using a MicropH 2002 pH meter equipped with a 52-07 pH electrode (Crison, Barcelona, Spain) [15,30].

Control groups included a plain medium (C−) and a medium supplemented with FOS (C+). Samples were promptly stored at −20 °C until subsequent analysis. This comprehensive methodology ensures controlled conditions for examining microbial dynamics and metagenomic outputs, offering crucial insights into gut microbiota interactions and their implications for host health [15,31,32].

### 2.4. Microbiota Assessment

#### 2.4.1. Genomic Material Extraction

All fermented samples were subjected to DNA extraction, which was performed using the NZY Tissue gDNA Isolation kit (NZYTech, Lisbon, Portugal), with slight modifications. Initially, pellets were washed with TE buffer (pH 8.0) and centrifuged at 4000× g for 10 min at 4 °C. Supernatants were discarded, and the pellets were treated with a freshly prepared lysozyme solution (10 mg/mL in TE buffer) for 2 h at 37 °C. After centrifugation, the supernatants were discarded, and subsequent steps followed the manufacturer’s protocol. The extracted DNA’s purity and concentration were assessed using a Thermo ScientificTM μDropTM Plate with a Thermo ScientificTM MultiskanTM FC Microplate Photometer (Thermo Fisher Scientific, Waltham, MA, USA).

#### 2.4.2. Metagenomics

The metagenomics analysis of 16S amplicons of these samples was conducted by Novogene Europe (Cambridge, UK), targeting the V4-V5 regions of 16S rRNA genes. CR amplification utilised specific primers with attached barcodes (5′-GTGCCAGCMGCCGCGGTAA-3′ and 5′-CCGTCAATTCCTTTGAGTTT-3′). PCR conditions involved an initial denaturation at 98 °C for 1 min, followed by 30 cycles of denaturation at 98 °C for 10 s, annealing at 50 °C for 30 s, elongation at 72 °C for 30 s, and a final extension at 72 °C for 5 min. PCR products were purified using a Universal DNA Purification kit (TianGen, Beijing, China). DNA concentration and purity were assessed spectrophotometrically, and integrity was confirmed by agarose gel electrophoresis. After quality validation, the extracted DNA was used for library preparation and subsequently shipped to Novogene Europe (Cambridge, UK) for sequencing and primary bioinformatic processing.

At Novogene, the samples underwent Illumina sequencing and initial data processing using the company’s validated metagenomic pipeline. This included quality assessment of raw reads, trimming of adapters and low-quality regions, denoising and chimera removal, followed by the generation of amplicon sequence variants (ASVs) through a workflow based on QIIME2 and DADA2. Taxonomic assignment was performed using a Naïve Bayes classifier trained on the SILVA (or Greengenes) reference database. As Novogene’s full internal pipeline is proprietary, only the final processed outputs were provided to us, namely the annotated taxonomic tables and the absolute and relative abundance matrices.

All downstream analyses, including data filtering, normalisation, diversity metrics, statistical testing, and ecological interpretation, were carried out by our team using R and the relevant microbiome analysis packages. This approach ensures a clear distinction between the laboratory and analytical steps performed internally and the sequencing and primary bioinformatic processing conducted externally.

### 2.5. Data-Driven Analysis

Microbial community data (relative-abundance tables) were imported into RStudio (R v4.3.1) and converted into a data frame. Dissimilarities among samples were quantified as Euclidean distances using the dist() function from the base stats package (stats::dist()), which does not require loading an external library. Hierarchical clustering was subsequently conducted with stats::hclust() using Ward’s method (ward.D2), which iteratively merges clusters to minimise the increase in within-cluster variance. Cluster relationships are displayed as a dendrogram [33].

(1) $$D\left(c_{1},c_{2}\right)=\frac{\left|c_{1}\right|\left|c_{2}\right|}{\left|c_{1}\right|+\left|c_{2}\right|}{\left|\left|c_{1}-c_{2}\right|\right|}^{2}$$

where *c*_1_ and *c*_2_ are the classes. This approach allowed for the analysis of the dissimilarities [16,17].

**Figure 2:**
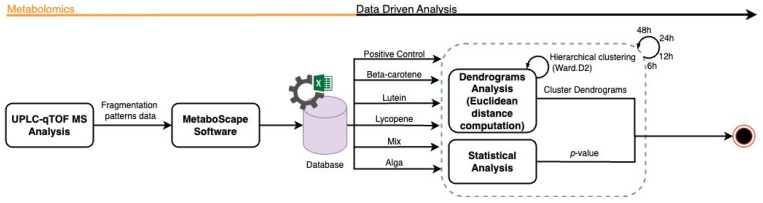
Flowchart representation of the metabolomics data analysis workflow.

To enhance interpretability, cluster boundaries were overlaid on the dendrograms using stats::rect.hclust(). In the global analysis (all samples), the dendrogram was partitioned into six clusters (k = 6). In analyses performed separately for each hourly time interval, dendrograms were partitioned into three clusters (k = 3), reflecting the dominant grouping structure apparent within each subset (Figure 2).

### 2.6. Statistical Analysis

Differences among multiple groups along the 48 h were tested via PERMANOVA (999 permutations) in R (version programming language version 4.3.1) using distance matrices computed via Bray–Curtis for abundances with the vegan::adonis2 R function, after confirming homogeneity of multigroup dispersions via betadisper R function. Statistical significance was set at *p* < 0.01, a stricter threshold widely adopted in microbiome research to minimise Type I errors and ensure robust inference [34].

## 3. Results

### 3.1. Heatmap Analysis

**Figure 3:**
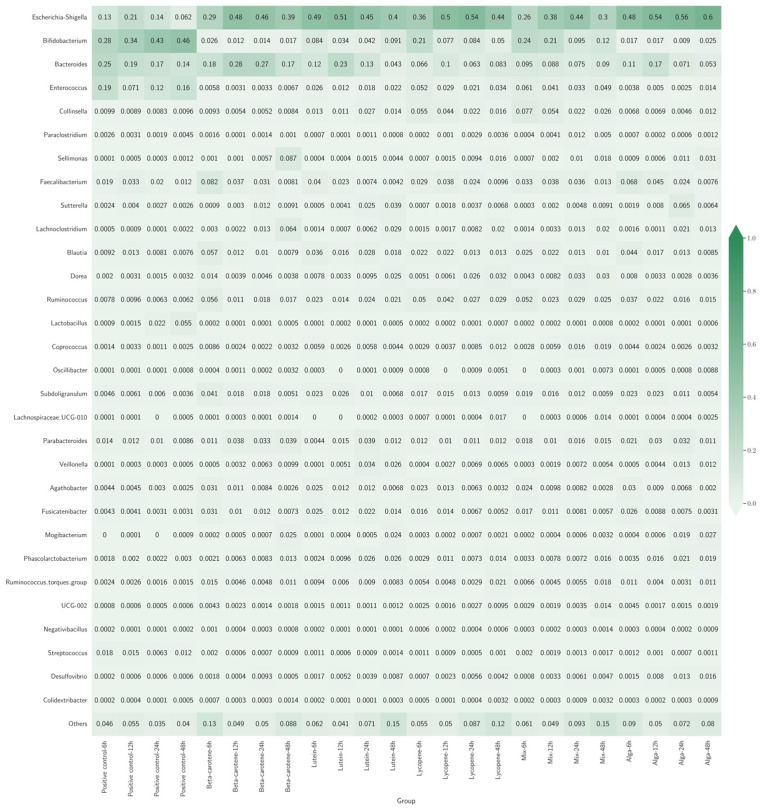
Heatmap with bacterial genera’s relative abundance (RA) across different sample groups. Each row represents a bacterial genus, and each column represents a different group. The colour intensity indicates how common each genus is in a group, with darker colours meaning higher abundance.

Figure 3 presents a heatmap illustrating the various bacterial genera’s relative abundance (RA) across different experimental groups. Each cell in the heatmap represents the proportion of a specific genus within each group, with colour intensity indicating the RA level. Darker shades correspond to higher abundances, while lighter shades indicate lower abundances.

Across all groups, *Escherichia-Shigella* was a prominent genus, representing a significant percentage of the classes’ composition, presenting the highest rate at 83.33% (20 out of 24) of the classes. In the β-carotene and Lycopene groups, *Escherichia-Shigella* exhibited a consistent increase over time, suggesting that these specific carotenoids may selectively enhance the growth of this genus. In contrast, the Lutein group saw a decrease in *Escherichia-Shigella* after an initial peak, indicating a transient effect that eventually stabilises or diminishes it. However, that trend does not happen for the positive control classes, with *Bifidobacterium* having the highest percentage of the composition of the classes.

The Mix and Alga groups demonstrated more balanced microbial communities compared to other groups. The Mix group maintained higher diversity with stable levels of *Bifidobacterium* and *Bacteroides* alongside *Escherichia-Shigella*. Similarly, the Alga group supported stable levels of beneficial bacteria like *Bifidobacterium* and *Lactobacillus*.

A summary of the gut bacterial taxa affected by carotenoids relative to the positive control, including trends over incubation time points, is provided in Table A1 of the Appendix A.

### 3.2. Cluster Dendrogram Analysis

**Figure 4:**
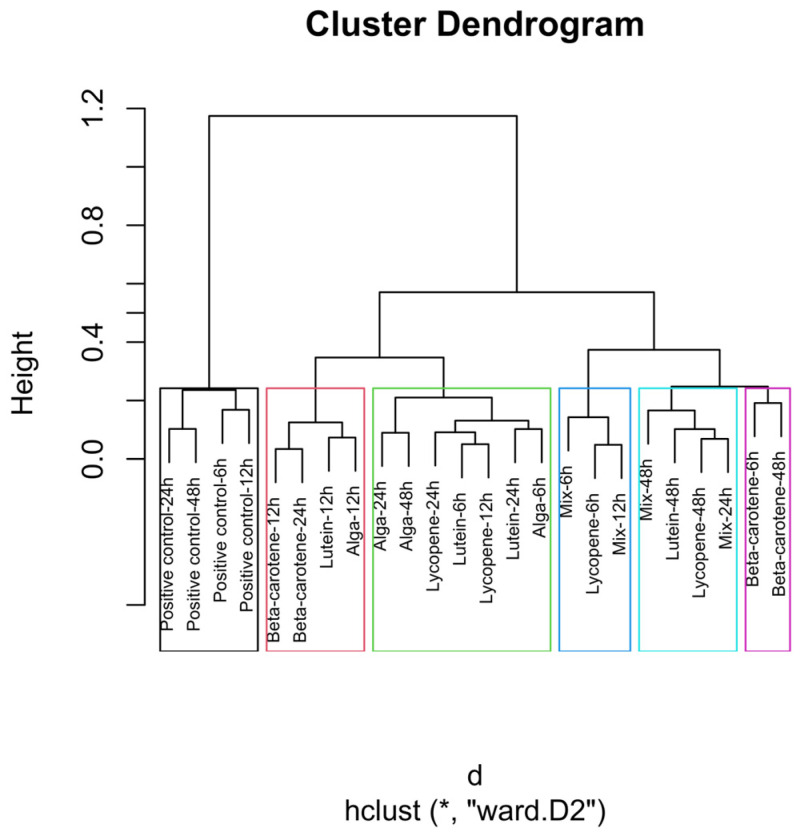
Global hierarchical clustering dendrogram of microbial compositions across different carotenoid treatments and time points. The green rectangle shows the highest number of samples with close distances. The blue rectangle shows proximity to the Mix 6 h, Lycopene 6 h, and Mix 12 h samples, with the last two presenting the same distance. The light blue indicates the close distance between the samples Mix 48 h, Lutein 48 h, Lycopene 48 h, and Mix 24 h, with the Lycopene at 48 h and the Mix at 24 h illustrating an equal distance. The black rectangle indicates a similar distance between the samples of β-carotene 6h and β-carotene 48h. Clusters identified by hierarchical analysis were statistically significant (*p* < 0.01, multiscale bootstrap resampling). The boxes representing the cluster groups have random colours, and “*” indicates distance.

**Figure 5:**
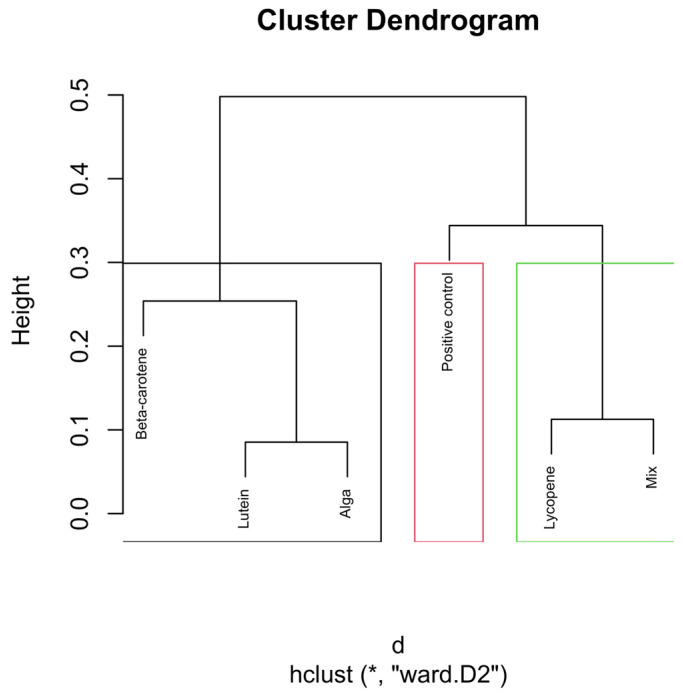
Hierarchical clustering dendrogram of microbial compositions across different carotenoid treatments at the 6 h time point. The red rectangle, “Positive control”, forms a distinct cluster, implying it isn’t similar to other groups. In the green rectangle, “Lycopene” and “Mix” form another cluster, indicating that they are more similar. Other groups like β-carotene, Lutein, and Alga, grouped separately on the left side, show similarities within themselves, distinct from the other clusters. Clusters identified by hierarchical analysis were statistically significant (*p* < 0.01, multiscale bootstrap resampling). The boxes representing the cluster groups have random colours, and “*” indicates distance.

**Figure 6:**
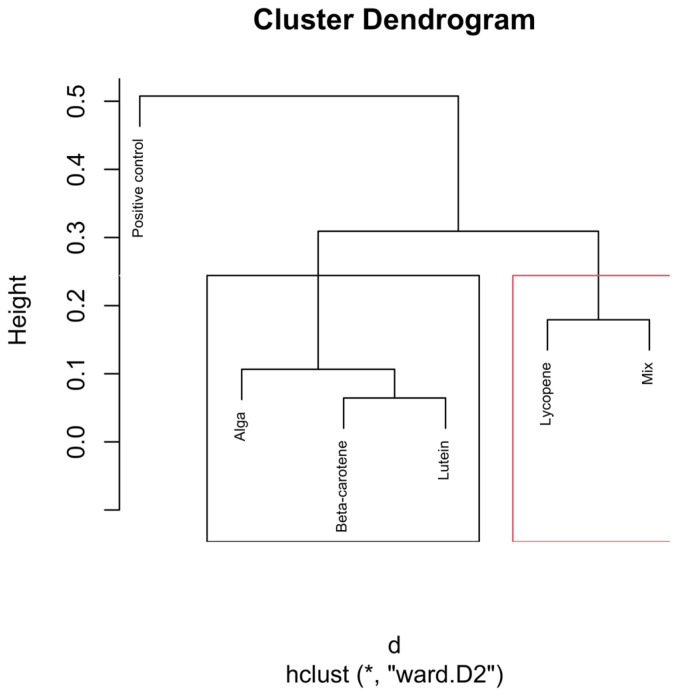
Hierarchical clustering dendrogram of microbial compositions across different carotenoid treatments at the 12 h time point. In the red rectangle, “Lycopene” and “Mix” form a cluster, indicating that they are more similar. Other groups like β-carotene, Lutein, and Alga, grouped separately on the left side, show similarities within themselves, distinct from the other clusters. Clusters identified by hierarchical analysis were statistically significant (*p* < 0.01, multiscale bootstrap resampling). The boxes representing the cluster groups have random colours, and “*” indicates distance.

**Figure 7:**
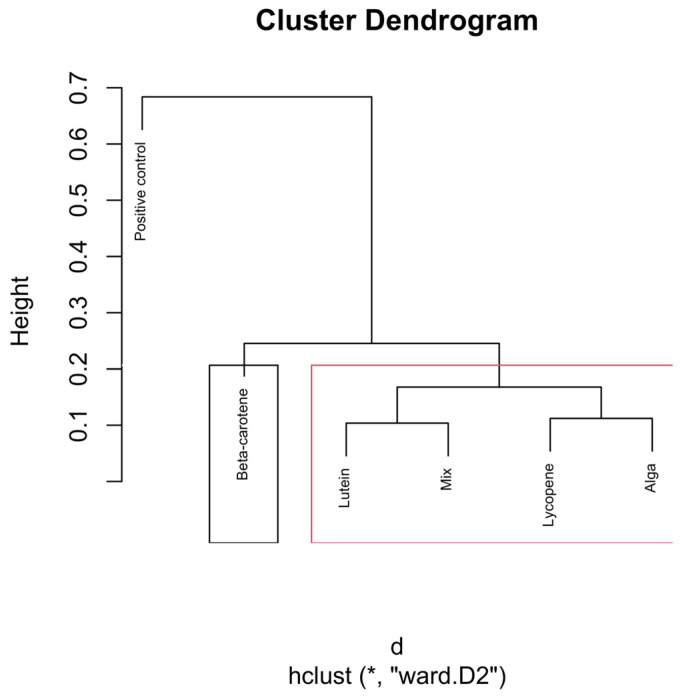
Hierarchical clustering dendrogram of microbial compositions across different carotenoid treatments at the 24 h time point. The black rectangle, “β-carotene”, forms a distinct cluster, implying it isn’t similar to other groups. In the red rectangle, “Lutein”, “Mix”, “Lycopene”, and “Alga” form another cluster, indicating that they are more similar within themselves, distinct from the other clusters. Clusters identified by hierarchical analysis were statistically significant (*p* < 0.01, multiscale bootstrap resampling). The boxes representing the cluster groups have random colours, and “*” indicates distance.

**Figure 8:**
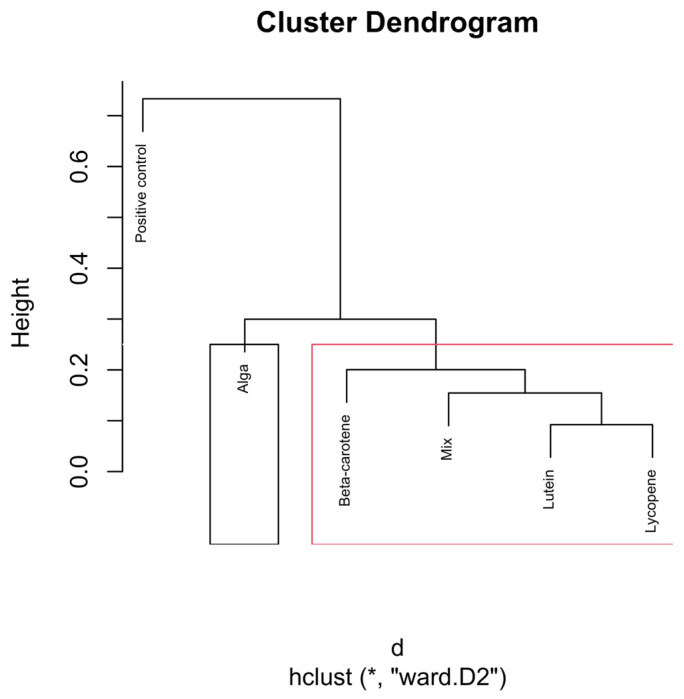
Hierarchical clustering dendrogram of microbial compositions across different carotenoid treatments at the 48 h time point. The black rectangle, “Alga”, forms a distinct cluster, implying it isn’t similar to other groups. In the red rectangle, β-carotene, Lutein, Lycopene, and Mix, grouped separately, show similarities within themselves, distinct from the other clusters. Clusters identified by hierarchical analysis were statistically significant (*p* < 0.01, multiscale bootstrap resampling). The boxes representing the cluster groups have random colours, and “*” indicates distance.

The dendrograms (Figure 4, Figure 5, Figure 6, Figure 7 and Figure 8) illustrate the hierarchical clustering of microbial compositions from various treatment groups subjected to different carotenoids and control conditions across time points (6, 12, 24, and 48 h). These analyses highlight the evolution of microbial communities during fermentation.

The positive control samples formed distinct clusters at 24 and 48 h, indicating unique microbial trajectories in the absence of carotenoids. The β-carotene group exhibited consistent clustering across time points, suggesting a stable influence on microbial composition. The Alga and Lutein groups displayed similar clustering at intermediate time points (12, 24, and 48 h), suggesting comparable microbial profiles. Lycopene and the Mix groups showed close clustering, particularly at 48 h, implying that the Mix effects were largely driven by lycopene.

## 4. Discussion

The combined analysis of Figure 3, Figure 4, Figure 5, Figure 6, Figure 7 and Figure 8 provides an integrated overview of how individual and combined carotenoids modulate gut microbiota composition and dynamics during in vitro fermentation. Collectively, these results demonstrate that carotenoid structural features, namely polarity, degree of conjugation, and matrix association, play a central role in shaping microbial responses over time. These characteristics influence community composition, acid tolerance, and ecological balance, resulting in distinct, treatment-specific microbial trajectories throughout fermentation. Although this study used a pooled faecal inoculum from Portuguese donors, similar taxonomic trends have been reported in in vitro fermentation studies conducted in other countries, where carotenoids and related dietary compounds also promoted increases in genera such as *Bifidobacterium* and *Bacteroides*, alongside shifts in acid-tolerant taxa. These consistencies suggest that the microbial responses observed here are not country-specific but reflect broader microbial dynamics associated with carotenoid exposure.

### 4.1. Heatmap Analysis

The heatmap (Figure 3) summarises treatment- and time-dependent changes in dominant bacterial genera. In the β-carotene group, *Escherichia-Shigella* showed a marked and progressive increase in relative abundance, reaching approximately a 10% rise at 48 h. This trend suggests that β-carotene selectively favours the growth or survival of this genus, potentially through modulation of fermentation conditions. Previously reported pH fluctuations during fermentation [15], see Appendix A, Table A2, particularly the early acidification phase, may have contributed to this pattern, as *Escherichia-Shigella* is known for its tolerance to acidic environments [35,36,37]. Concurrently, other bacterial genera in this group experienced a general decline in RA, indicating a competitive displacement or suppression by *Escherichia-Shigella* under the influence of β-carotene.

In the Lutein group, the initial bacterial composition at 6 h was characterised by moderate levels of both *Escherichia-Shigella* and *Bacteroides*. However, the dynamics within this group over time revealed distinct fluctuations. *Escherichia-Shigella* and *Bacteroides* peaked at the 12 h and 24 h time points, indicating a temporary surge in their relative abundances. By the 48 h mark, *Escherichia-Shigella* decreased by 9%, and *Bacteroides* saw a 7.7% reduction. The fluctuations in *Escherichia-Shigella* and *Bacteroides* in the Lutein group can be linked to changing fermentation conditions, including pH variation and nutrient availability [15]. This suggests that pH variations played a crucial role in their population dynamics (Table A2).

The Lycopene group presented a different pattern than that reported earlier for the β-carotene group. Initially, *Escherichia-Shigella* exhibited a moderate presence at the 6 h time point. However, unlike the Lutein group, lycopene promoted a sustained increase in *Escherichia-Shigella* over time, with an 8% rise in RA by the 48-h mark. This response suggests that lycopene created a microbial environment that consistently favours acid-tolerant taxa over time [15].

The Mix group combined three different carotenoids and demonstrated a more balanced microbial composition across the time points. At 6 h, the RA of *Escherichia-Shigella* was relatively low, with *Bifidobacterium* and *Bacteroides* showing higher abundances. Unlike the individual carotenoid groups, the Mix group exhibited a more gradual increase in *Escherichia-Shigella*, alongside a sustained presence of *Bifidobacterium* and *Bacteroides* throughout the 48 h [15]. This stability suggests potential synergistic effects among carotenoids, promoting microbial diversity and preventing the dominance of a single genus.

The Alga group, featuring carotenoids derived from the red marine alga *Osmundea pinnatifida*, showed unique trends in bacterial composition. At the 6 h mark, *Escherichia-Shigella* was present alongside higher levels of *Bifidobacterium* and *Lactobacillus* compared to other groups. Over time, there was a slight increase in *Escherichia-Shigella*, but unlike the other groups, the RA of *Bifidobacterium* and *Lactobacillus* did not decrease significantly [15]. The persistence of beneficial, acid-tolerant genera suggests that algal carotenoids support a more stable and health-associated microbial community.

These group-specific observations reveal the nuanced effects of different carotenoids on gut microbiota composition, highlighting treatment-specific and time-dependent microbial trajectories. The varying responses of *Escherichia-Shigella*, *Bifidobacterium*, *Bacteroides*, and other genera underscore the complexity of microbial interactions influenced by specific dietary components.

Beyond the taxonomic shifts observed across treatments, the metabolic implications of these microbial changes are particularly relevant to NCDs. The promotion of beneficial genera such as *Bifidobacterium*, *Lactobacillus*, and *Bacteroides*, together with the stimulation of short-chain fatty acid production (notably butyrate, acetate, and propionate) as discussed in Rocha et al. (2024) [15], aligns with microbial profiles associated with reduced chronic inflammation, improved glucose homeostasis, and enhanced intestinal barrier integrity. Conversely, the modulation of potentially proinflammatory taxa such as *Escherichia-Shigella* highlights the ability of carotenoids to regulate dysbiosis-associated microbial patterns. Together, these effects suggest a role for carotenoid-driven microbial restructuring in mitigating chronic metabolic and inflammatory disorders.

### 4.2. Relative Abundance—Cluster Dendrogram Analysis

The global clustering dendrogram (Figure 4) further confirms the distinct microbial environments established by each treatment. The positive control samples, which did not receive any carotenoid treatment, formed a distinct cluster, particularly at the 24 h and 48 h time points. This clustering indicates that, in the absence of carotenoids, microbial composition follows its natural fermentation trajectory.

The β-carotene group exhibited a clear and consistent clustering pattern, particularly at the 6, 12, and 48 h time points. This consistency suggests that β-carotene had a stable and reproducible effect on the microbial composition throughout fermentation. These results indicate a robust treatment-specific restructuring of the microbial community.

The Alga group, which includes carotenoids derived from *O. pinnatifida*, and the Lutein group displayed clustering patterns that were closely linked, particularly at the 12, 24, and 48 h time points. This close clustering suggests that these two treatments may promote similar microbial communities, possibly reflecting comparable biochemical or matrix-associated characteristics.

The Lycopene and Mix groups also showed a notable clustering pattern, especially at the 48-h mark. This proximity suggests that lycopene largely drives the microbial response within the mixed formulation.

This analysis underscores the role of carotenoids in modulating gut microbiota, with each treatment creating distinct microbial environments that evolve throughout the fermentation process.

The dendrogram in Figure 5 shows strong differentiation between treatments in the early fermentation phase. The β-carotene group forms a distinct and isolated cluster in the dendrogram, indicating that the microbial composition under the influence of β-carotene at the 6 h mark significantly differs from that of the other treatment groups (*p* < 0.01). This indicates a rapid and specific microbial response to β-carotene supplementation.

The Lutein and Alga groups cluster closely together in the dendrogram, suggesting that these two treatments have similar effects on the microbial composition at the 6 h time point.

The positive control group, which did not receive any carotenoid treatment, forms a separate cluster, distinct from the β-carotene and the Lutein/Alga clusters. This distinct clustering underscores carotenoids’ significant role in altering the microbial composition, even at an early fermentation stage.

The Lycopene and Mix groups exhibit close clustering in the dendrogram, particularly at the 6 h mark, suggesting that these two treatments produce similar microbial compositions during the early stages of fermentation.

The dendrogram presented in Figure 6 illustrates the hierarchical clustering of microbial compositions at the 12 h time point across different carotenoid treatments.

The positive control group forms a distinct and separate branch from all the other groups. This clear separation suggests that the microbial composition in the absence of carotenoids follows a different trajectory during fermentation. The distinct clustering indicates that carotenoid supplementation significantly impacts (*p* < 0.01) the microbial environment, differentiating it from the natural progression observed in the control group.

The Lycopene and Mix groups cluster closely together, forming a distinct branch separate from the other carotenoid treatments. This reinforces the dominant contribution of lycopene within the mixed formulation at this point.

The Alga, β-carotene, and Lutein groups cluster together on a separate branch from the Lycopene and Mix groups, but with some internal differentiation. The tight clustering of β-carotene and lutein indicates that these two carotenoids may induce similar shifts in the microbiota, possibly due to their shared biochemical properties or similar effects on the fermentation environment.

Consequently, the hierarchical clustering at the 12 h mark reflects different carotenoid treatments’ influence on the gut microbiota.

The dendrogram in Figure 7 reveals distinct clustering patterns in the microbial community structures influenced by the carotenoid treatments at the 24 h time point.

Like the previous time points, the positive control group continues to form a separate branch from all carotenoid-treated groups. The clear separation indicates that the microbiota without carotenoids follows a different fermentation trajectory. This indicates that carotenoid-driven effects become more pronounced with fermentation time.

The β-carotene group forms a unique and independent cluster, separate from the other carotenoid treatments. This suggests that β-carotene exerts a distinct influence on the gut microbiota, likely favouring specific bacterial genera or metabolic pathways that differ from those influenced by the other carotenoids.

The Lutein, Mix, Lycopene, and Alga groups cluster more closely together, forming a sub-branch, indicating that these carotenoids promote a similar microbial environment. Lutein shows a closer association with the Mix group, suggesting shared microbial outcomes. Lycopene clusters closely with Alga (*O. pinnatifida*), suggesting a shared influence on the microbial environment.

The dendrogram in Figure 8 shows clear clustering patterns that reflect the distinct effects of various carotenoid treatments after extended fermentation.

The positive control remains isolated, forming its separate cluster, indicating that microbial communities in the absence of carotenoid treatments continue to follow a divergent trajectory.

The Alga group clusters closely with β-carotene at the 48 h mark. This shift suggests the convergence of microbial communities after prolonged exposure.

β-Carotene continues to demonstrate a distinct clustering pattern and now shares a closer relationship with the mix of carotenoids. This indicates convergence toward a stable microbial configuration.

Further down the dendrogram, Lutein and Lycopene cluster together, demonstrating a distinct relationship between these two carotenoids by the 48 h time point.

Taken together, the dendrogram analyses demonstrate that carotenoids do not merely induce transient microbial fluctuations but instead promote structured, treatment-specific ecological trajectories that persist throughout fermentation. Such stability and directionality are highly relevant to human health, as long-term microbial configurations play a central role in modulating inflammatory tone, metabolic regulation, and mucosal integrity, key processes underlying NCDs [38,39,40]. Overall, carotenoid-driven clustering patterns indicate a capacity to counteract dysbiosis-associated microbial trajectories.

### 4.3. Clinical and Nutritional Implications

The modulation of gut microbiota by carotenoid carriers has clear translational relevance for nutrition and clinical applications. The observed increases in beneficial genera (*Bifidobacterium*, *Lactobacillus*, and *Bacteroides*) and short-chain fatty acid (SCFA) production suggest that dietary carotenoids could reinforce intestinal homeostasis and reduce risk factors for chronic metabolic and inflammatory diseases [15]. SCFAs support gut barrier function, modulate systemic inflammation, and contribute to energy metabolism, linking microbial shifts to improved glucose homeostasis, lipid profiles, and immune regulation [22,23,25,41,42].

From a dietary perspective, carotenoid-rich foods, such as fruits, vegetables, and certain algae, may serve as practical interventions to promote a health-associated microbiota [7,10,11,12]. Differences between individual carotenoids and their combinations indicate opportunities to tailor interventions to specific microbial outcomes. For example, the Mix and Alga groups’ ability to maintain microbial diversity while moderating potentially proinflammatory taxa could guide the development of functional foods designed to enhance microbial resilience and metabolic health.

Clinically, these results support carotenoid-enriched interventions for populations at risk of obesity, type 2 diabetes, and cardiovascular disease [10,11,12]. By modulating gut microbiota composition and activity, carotenoids could complement personalised nutrition strategies, taking into account individual microbiome profiles. The sustained microbial shifts observed suggest that regular consumption of carotenoid-rich foods may contribute to durable improvements in gut ecosystem stability and inflammatory control.

In the context of human physiology, it is also important to consider that gut microbiota composition varies substantially across the lifespan and along the gastrointestinal tract. Aging is associated with a progressive reduction in microbial diversity, a decline in beneficial taxa such as *Bifidobacterium*, and alterations in SCFA production, often accompanied by increased low-grade inflammation and metabolic dysregulation [43,44,45,46].

These age-related shifts may influence the responsiveness of the gut ecosystem to dietary interventions, including carotenoid intake. Similarly, microbial composition differs along the digestive tract due to gradients in pH, oxygen availability, bile acids, and transit time, with the colon hosting the highest microbial density and fermentative activity [47,48,49]. As the present in vitro fermentation model primarily reflects colonic conditions, the observed microbial modulation by carotenoids is most relevant to the large intestine, where carotenoid-derived metabolites and microbial fermentation products are expected to exert their major physiological effects. Acknowledging these spatial and age-related variations strengthens the translational interpretation of carotenoid-driven microbiota modulation while highlighting the need for future in vivo studies across different age groups.

For functional food formulation, carotenoid type, combination, and bioaccessibility should be considered to maximise prebiotic-like effects on the microbiota [6,50]. Blends mimicking the Mix or Alga groups could preserve microbial diversity, enhance SCFA production, and support intestinal health. Potential synergistic effects support the use of mixed carotenoid formulations or whole-food matrices over isolated compounds.

Overall, these findings provide mechanistic and translational support for incorporating carotenoid-driven microbial modulation into dietary strategies, functional foods, and nutraceuticals aimed at promoting gut health and reducing chronic disease risk.

## 5. Conclusions

This study provides bioinformatic insights into the influence of carotenoids on gut microbiota composition and metabolic functionality, supporting their role as dietary modulators of intestinal health. By employing metagenomic and robust bioinformatic analyses, we observed significant microbial shifts in response to carotenoid treatments, including the enhancement of beneficial taxa such as *Lachnospiraceae*, *Bifidobacterium*, and *Bacteroides*, alongside reductions in potentially harmful genera like *Enterococcus* and *Streptococcus*. Notably, the combined carotenoid treatment (Mix group) exhibited a synergistic effect, fostering a balanced and resilient microbial community and a stabilising influence on microbial diversity over time.

Hierarchical clustering analyses further revealed that carotenoids induce distinct, treatment-specific and time-dependent microbial trajectories, indicating that their effects extend beyond transient microbial fluctuations to promote structured and sustained ecological patterns throughout fermentation. Taken together, these findings suggest potential implications for inflammatory regulation, metabolic homeostasis, and mucosal integrity, key factors in NCD prevention.

Overall, our findings underscore the potential of carotenoids as functional dietary components capable of modulating gut microbiota composition and activity. These properties position carotenoids as promising candidates for dietary strategies aimed at improving gut health and mitigating risks associated with metabolic and inflammatory disorders. Future research should focus on elucidating the molecular mechanisms underlying these interactions and evaluating the long-term benefits of carotenoid-rich diets in diverse populations to better support human health.

## Figures and Tables

**Table 1:** Recovery indexes (%) for plain β-carotene and plain lutein at various gastrointestinal tract (GIT) sampling phases.

Carotenoid Group	GIT Phase	Recovery Index (%)
β-carotene	IM	0.4
Lutein	SSP	0.02
	SGP	0.04
	SIP	0.27

Abbreviations: IM, intestinal microbiota; SSP, simulated salivary phase; SGP, simulated gastric phase; SIP, simulated intestinal phase.

## Data Availability

The original contributions presented in the study are included in the article; further inquiries can be directed to the corresponding author.

## Abbreviations

The following abbreviations are used in this manuscript:

IM	Intestinal Microbiota
NCD	Non-Communicable Disease
OA	Organic Acid
PBS	Phosphate-Buffered Saline
FOS	Fructooligosaccharides
TSB	Tryptone Soya Broth
RPS	Reduced Physiological Salt Solution
SSF	Simulated Salivary Fluid
SGF	Simulated Gastric Fluid
SIF	Simulated Intestinal Fluid
OA(s)	Organic Acid (s)
DNA	Deoxyribonucleic Acid
RNA	Ribonucleic Acid
UPLC-qTOF-MS/MS	Ultra-Performance Liquid Chromatography Coupled to Quadrupole Time-of-Flight Tandem Mass Spectrometry
ESI	Electrospray Ionisation
DAD	Diode-Array Detector
RA	Relative Abundance
α-diversity/β-diversity	Alpha and Beta Diversity

## Appendix A

**Table A1:** Summary of gut bacterial taxa affected by carotenoid supplementation relative to the positive control. Arrows indicate the overall trend in RA across incubation time points (↑ increase; ↓ decrease; = no consistent change).

Bacterial Taxon	Effect of Carotenoids
*Bifidobacterium*	=
*Lactobacillus*	=
*Faecalibacterium*	=
*Lachnospiraceae* spp.	=
*Agathobacter*	=
*Fusicatenibacter*	=
*Subdoligranulum*	=
*Bacteroides*	=
*Parabacteroides*	=
*Escherichia–Shigella*	↑
*Enterococcus*	↓
*Collinsella*	=
*Veillonella*	=
*Streptococcus*	↓
*Sutterella*	=
*Lachnoclostridium*	=
*Blautia*	=
*Dorea*	=
*Ruminococcus*	↓
*Coprococcus*	=
*Phascolarctobacterium*	=
*Desulfovibrio*	=

**Table A2:** pH values measured at 0, 6, 12 and 24 h for all experimental conditions, including negative and positive controls, individual carotenoids (β-carotene, lycopene, lutein), their mixture, and the algal extract. Values are expressed as mean ± standard deviation.

Sample/Time Point	0 h	6 h	12 h	24 h
**Negative Control**	6.72 ± 0.06	6.01 ± 0.02	6.65 ± 0.01	6.15 ± 0.02
**Positive Control**	6.74 ± 0.04	4.57 ± 0.07	6.09 ± 0.01	3.62 ± 0.01
**Beta-Carotene**	6.95 ± 0.01	6.01 ± 0.01	6.61 ± 0.01	6.21 ± 0.02
**Lycopene**	6.86 ± 0.01	5.92 ± 0.01	6.67 ± 0.03	6.16 ± 0.01
**Lutein**	6.86 ± 0.01	5.9 ± 0.04	6.63 ± 0.02	6.15 ± 0.04
**Mix**	6.93 ± 0.01	5.92 ± 0.05	6.68 ± 0.03	6.12 ± 0.01
**Alga**	7.19 ± 0.01	6.11 ± 0.04	6.69 ± 0.04	6.28 ± 0.03
